# Imaging-Based Molecular Interaction Between Src and Lamin A/C Mechanosensitive Proteins in the Nucleus of Laminopathic Cells

**DOI:** 10.3390/ijms252413365

**Published:** 2024-12-13

**Authors:** Stefania Petrini, Giulia Bagnato, Michela Piccione, Valentina D’Oria, Valentina Apollonio, Marco Cappa, Claudia Castiglioni, Filippo Maria Santorelli, Teresa Rizza, Rosalba Carrozzo, Enrico Silvio Bertini, Barbara Peruzzi

**Affiliations:** 1 Microscopy Core Facility, Research Center, Bambino Gesù Children’s Hospital, IRCCS, 00146 Rome, Italy; stefania.petrini@opbg.net (S.P.); michela.piccione@opbg.net (M.P.); valentina.doria@opbg.net (V.D.); v.apollonio@hotmail.com (V.A.); 2 Bone Pathophysiology Research Unit, Bambino Gesù Children’s Hospital, IRCCS, 00146 Rome, Italy; giulia.bagnato@uniroma1.it; 3 DAHFMO–Unit of Histology and Medical Embryology, Sapienza University of Rome, 00161 Rome, Italy; 4 Research Unit for Innovative Therapies in Endocrinopathies, Bambino Gesù Children’s Hospital, IRCCS, 00146 Rome, Italy; marco.cappa@opbg.net; 5 Department of Neurology, Clínica Meds and National Rehabilitation Institute Pedro Aguirre Cerda, Santiago 8460000, Chile; castiglionic@gmail.com; 6 Molecular Medicine for Neurodegenerative and Neuromuscular Diseases Unit, Fondazione Stella Maris, IRCCS, 56128 Pisa, Italy; filippo3364@gmail.com; 7 Laboratory of Medical Genetics, Translational Cytogenomics Research Unit, Bambino Gesù Children’s Hospital, IRCCS, 00146 Rome, Italy; teresa.rizza@opbg.net (T.R.); rosalba.carrozzo@opbg.net (R.C.); 8 Research Unit of Muscular and Neurodegenerative Disorders, Bambino Gesù Children’s Hospital, IRCCS, 00146 Rome, Italy; enricosilvio.bertini@opbg.net; 9 Istituto di Istologia ed Embriologia, Università Cattolica del Sacro Cuore, 00168 Rome, Italy

**Keywords:** laminopathies, mechanosensitive proteins, nuclear envelope proteins, lamin A/C, Src tyrosine kinase, STED-microscopy, FLIM/FRET analysis, fluorochrome lifetime

## Abstract

Laminopathies represent a wide range of genetic disorders caused by mutations in gene-encoding proteins of the nuclear lamina. Altered nuclear mechanics have been associated with laminopathies, given the key role of nuclear lamins as mechanosensitive proteins involved in the mechanotransduction process. To shed light on the nuclear partners cooperating with altered lamins, we focused on Src tyrosine kinase, known to phosphorylate proteins of the nuclear lamina. Here, we demonstrated a tight relationship between lamin A/C and Src in skin fibroblasts from two laminopathic patients, assessed by advanced imaging-based microscopy techniques. With confocal laser scanning and Stimulated Emission Depletion (STED) microscopy, a statistically significant higher co-distribution between the two proteins was observed in patients’ fibroblasts. Furthermore, the time-domain fluorescence lifetime imaging microscopy, combined with Förster resonance energy transfer detection, demonstrated a decreased lifetime value of Src (as donor fluorophore) in the presence of lamin A/C (as acceptor dye) in double-stained fibroblast nuclei in both healthy cells and patients’ cells, thereby indicating a molecular interaction that resulted significantly higher in laminopathic cells. All these results demonstrate a molecular interaction between Src and lamin A/C in healthy fibroblasts and their aberrant interaction in laminopathic nuclei, thus creating the possibilities of new diagnostic and therapeutic approaches for patients.

## 1. Introduction

Lamins, the main components of the nuclear lamina, are type V intermediate filament proteins that polymerize to form a highly organized meshwork between the inner nuclear membrane (INM) and the chromatin [1]. Lamins are essential for the maintenance of nuclear structure and mechanics, gene regulation, chromatin organization, telomere homeostasis, DNA replication, and damage repair [1,2,3]. Lamins are grouped into A-type and B-type depending on their structural and biochemical properties. A-type lamins bind to B-type lamins and to several structural proteins, including the integral INM protein emerin, the outer nuclear membrane (ONM) proteins’ nesprins, lamina-associated polypeptide 2 isoform alpha (LAP2α), NUP153, SUN-domain-containing proteins, nuclear actin, and protein 4.1R, thus forming a structural network essential for nuclear integrity and nucleo-cytoskeletal coupling [4]. In mammals, the *LMNA* gene gives rise to lamins A and C via alternative RNA splicing, and they are expressed in a tissue- and development–specific manner, whereas B-type lamins encoded by the *LMNB1* (lamin B1) and *LMNB2* (lamins B2 and B3) genes are ubiquitously expressed [5,6,7,8].

*LMNA* mutations cause a heterogeneous spectrum of rare human diseases commonly known as “laminopathies”, involving different tissues, such as striated muscle (including cardiac muscle, causing cardiomyopathies), adipose tissue (lypodystrophy syndromes), peripheral nerve (peripheral neuropathy), or multiple systems with features of accelerated aging [9]. The most severe laminopathies are progeroid syndromes, including the premature aging disease, such as Hutchinson-Gilford progeria syndrome (HGPS-OMIM 176670), Atypical Werner’s syndrome (ORPHA79474), restrictive dermopathy (OMIM 619793), and mandibular acral dysplasia (OMIM 248370). The striated muscle laminopathies include disorders such as Emery-Dreifuss muscular dystrophy (EDMD-OMIM 181350) or limb-girdle muscular dystrophy type 1B, where cardiac muscle involvement represents the common feature coexisting without or with skeletal muscle disease. In addition, the EDMD may also occur as a result of mutations in the *EMD* gene encoding emerin [10]. While canonical Werner syndrome is a prototypical segmental progeroid syndrome characterized by multiple features consistent with accelerated aging, and is caused by null mutations of the *WRN* gene and the atypical form derived by the mutation on the *LMNA* gene, and follows an autosomal dominant pattern of inheritance. Atypical Werner’s syndrome shows accelerated aging characterized by short stature, thinning/graying of hair, a “bird-like” facial appearance, skin atrophy, lipodystrophy, and myopathy, along with other age-related disorders, such as osteoporosis and atherosclerosis. Compared to the canonical form, it has an earlier age of onset (early 20 s or earlier) and a more rapid rate of progression [11]. With regard to the *LMNA*-related congenital muscular dystrophy, this is a condition that primarily affects muscles used for movement (skeletal muscles) and is characterized by prominent axial hypotonia, predominantly proximal muscle weakness in upper limbs and distal in lower limbs, joint contractures, spinal rigidity, and progressive respiratory insufficiency, in the presence of moderately elevated serum creatine kinase. Cardiac arrhythmias and sudden death have also been reported [12].

Previous reports have highlighted the pivotal role of post-translational modifications of lamins in maintaining their proper functions, distributions, and structural properties, such as farnesylation of the cysteine residue of the C-terminal CAAX motif, serine or tyrosine phosphorylation, SUMOylation, acetylation, O-Glc-NAcylation, and ubiquitination [13,14].

Src, the most representative member of the Src family of tyrosine kinases (SFKs), is involved in several cellular processes, such as cell proliferation, migration, and cell response to mechanical stimulation [15,16]. It is worth noting that, in addition to the most studied functions in the cytoplasmic compartment, Src kinases exert several roles in the nucleus, acting as kinase for targeted nuclear proteins and as cooperating protein in multi-complexes involved in regulating gene expression [17].

Given this function of Src as a kinase of nuclear envelope proteins, as lamin A and emerin [18,19,20], and that lamin A activity is regulated by phosphorylation [21], in this work, we focused on the relationship between lamin A/C and Src proteins in healthy and laminopathic fibroblasts at the nanoscale level using confocal and super-resolution STED microscopy; we used advanced imaging tools, such as FRET after photobleaching (FRET-AB) and FLIM-FRET analyses to investigate their intermolecular distances and interactions.

FRET imaging is a non-radiative method of energy transfer from a donor fluorophore in the excited state to an acceptor fluorophore through long-range dipole–dipole interactions. However, FRET can occur only when donor and acceptor molecules are in close proximity (typically 1–10 nm) and the emission spectrum of a donor fluorophore significantly overlaps (>30%) the absorption spectrum of an acceptor [22].

FLIM is a highly useful tool that allows for spatially resolving the lifetimes of one or more fluorophores. The fluorescence lifetime is the time expressed in pico- or nano-seconds in which the fluorophore remains in the excited state before returning to the ground state, and it is sensitive to environmental conditions (temperature, pH, oxygen content, ion concentration, etc.) and to excited state reactions, such as FRET, whereas it is not influenced by probe concentration, photobleaching, or internal settings (laser intensity, detector gain) of the instrument [23,24]. Because FRET reduces the donor lifetime in proportion to the energy transfer efficiency, FLIM analysis can be used for quantitative FRET detection. As a result, the combination of FRET and Fluorescence Lifetime (FRET-FLIM) methods provides high spatial (nanometer) and temporal (nanosecond) resolution by monitoring the change in donor lifetime in the presence and absence of an acceptor. FRET detection by time-domain FLIM has been applied to the characterization of protein–protein interactions with high spatial and temporal specificity (e.g., clustering), in the study of conformational changes, and in the analysis of binding sequences [22,24].

In this study, we provide evidence of the differential Src distribution in the dermal fibroblast nuclei, and its close relationship with lamin A/C, thereby strengthening the potential role of the Src–lamin A/C binding in pathogenesis and in the altered mechanosensitive processes linked to laminopathies.

## 2. Detailed Case Description

### 2.1. Patients’ Biological Samples

Two genetically confirmed *LMNA* patients have been the objective of this manuscript, followed by clinicians of Bambino Gesù Children’s Hospital, Rome, Italy (Patient 1) and at the National Rehabilitation Institute (INRPAC) in Santiago, Chile (Patient 2), respectively. Biological samples, including blood and skin biopsies, were obtained during the diagnostic investigation after obtaining signed informed consent. DNA was obtained from circulating leukocytes using standard procedures. Aged- and sex-matched controls of skin fibroblasts were also used. The study has been conducted according to the guidelines established in the Declaration of Helsinki. The collected data were stored in individual clinical records. Considering the retrospective nature of the analysis, the current study did not require the approval of the local ethics committee according to current legislation, but a notification was sent. Data were retrospectively analyzed in line with personal data protection policies.

### 2.2. Patients

#### 2.2.1. Patient 1

The patient was born in 1985. At birth, this lady was surgically operated on for an umbilical hernia with expulsion of viscera. At the age of 8, she was reported to have acrogeria with lipodystrophy; later, at the age of 18, she was diagnosed with type II diabetes mellitus, and at the age of 25, she was admitted to the Endocrinology Department with the diagnosis of acrogeria to carry out laboratory control tests. The physical examination showed that she was in good general condition, having a weight of 39.4 kg (<3rd centile), height of 150 cm (3rd centile), HR of 79 bpm, and BP of 108/63 mmHg. She had characteristic facies with micrognathia, a pinched face, an “owl-eyed” appearance, and a beaked nose with marbled and thin skin. Diffuse hypertrichosis was observed, especially on the face and dorsal region, as well as hypotrophy of the muscle masses. Her abdomen was treatable; there was an umbilical hernia and no organomegaly. Abdomen ultrasound imaging only showed that her liver had a slightly heterogeneous echo structure but without focal lesions, and a cardiological examination with EKG and echocardiography showed no abnormalities. A laboratory examination showed normal CK and transaminases and an abnormal glycemic curve with slightly increased insulin. At a subsequent control, at the age of 26, she showed impaired glucose tolerance, increased triglycerides, and mitral valvular insufficiency, and two years later, a liver echo showed that it increased in size with a diffuse and homogeneous increase in echogenicity, suggesting hepatic steatosis. The patient was discharged with the diagnosis of type II diabetes and persistent valvular mitral insufficiency. The patient passed away at the age of 30 due to mandibular sarcoma that invaded surrounding tissues (tongue and palate). Mutational analysis by direct Sanger sequencing of the *LMNA* gene (NM_170707.4), highlighted a heterozygous likely pathogenetic mutation c.1733A>T (p.Glu578Val) described elsewhere [25], confirming the clinical presentation of a progeroid syndrome, specifically atypical Werner’s syndrome.

#### 2.2.2. Patient 2

The patient was the youngest of two siblings, born to non-consanguineous parents. She developed normally until 14 months. After she started walking, she began to have a waddling gait, fell frequently, and had difficulty getting up from the floor. Progressive proximal upper and lower limb weakness and serum creatine kinase levels were tenfold above the noted normal range. A muscle biopsy showed dystrophic changes. She lost the ability to walk at 31 months old and developed severe contractures in her elbows, wrists, hips, knees, elbows, and ankles. Over the years, she also developed hyperlordosis and a rigid spine. At the age of 15 years, she needed nocturnal non-invasive ventilation due to restrictive ventilatory insufficiency. When she was 16, she received an implantable defibrillator because of recurrent ventricular extrasystoles and a first-degree atrioventricular (AV) block. Sadly, she passed away at the age of 19 due to secondary heart failure. Mutational analysis via direct sequencing of the *LMNA* gene (NM_170707.4) pointed out the heterozygous missense variant c.745C>T (p.Arg249Trp), previously described in other patients affected by congenital muscular dystrophy [26].

### 2.3. Cell Cultures

Skin fibroblasts from laminopathic patients and two controls were grown in Dulbecco’s modified minimum essential medium (D-MEM), supplemented with 10% fetal bovine serum (Life Technologies, Segrate, Italy; a part of Thermo Fisher Scientific, Waltham, MA, USA), and 1% penicillin and streptomycin (Life Technologies, Segrate, Italy; a part of Thermo Fisher Scientific).

### 2.4. Immunofluorescence

In total, 5 × 10^3^ cells were seeded into plastic-chambered glass microscope slides (BD Falcon), grown until sub-confluence, and fixed with 4% paraformaldehyde in PBS for 10 min followed by PBS/Triton 0.1% for 5 min. The following antibodies were used according to manufacturer instructions: primary rabbit anti-Src (sc-19) and mouse anti-lamin A/C (sc-7292) antibodies, purchased from Santa Cruz Biotechnology (Temecula, CA, USA); they were diluted in 1% PBS/BSA overnight, at 4 °C. Secondary goat immunoglobulins conjugated with Alexa Fluor 488 and 594 dyes (Life Technologies) have been used for confocal microscopy acquisitions. Slides were mounted with PBS/glycerol 1:1. In parallel experiments, primary anti-Src and lamin A/C were revealed using goat anti-rabbit and goat anti-mouse conjugated to STAR Orange and STAR Red dyes (Abberior, Göttingen, Germany), respectively, and, after PBS washes, slides were mounted using the mount solid antifade (Abberior) mounting medium and #1.5 thickness coverslips.

### 2.5. Confocal Microscopy Imaging and Data Analysis

Confocal microscopy was performed on a Leica TCS-SP8X laser-scanning confocal microscope (Leica Microsystems, Mannheim, Germany) equipped with a tunable white light laser (WLL) source, a 405 nm diode laser, 3 internal spectral detector channels (PMTs), including 2 dedicated to single molecule detection (SMD), and 2 internal spectral detector channels (HyD) GaAsP. Sequential confocal images were acquired using HC PL APO 40x/oil immersion (1.30 numerical aperture, NA, Leica Microsystems) and 63x/oil immersion objectives (1.40 NA, Leica Microsystems). The mean fluorescence intensities (MFI) of Src and lamin A/C were manually measured in each nucleus, and then calculated using MetaMorph 7.8 (Molecular Devices, San Jose, CA, USA) software. From seven digital images (40x magnification), a number of nuclei >80 was randomly selected and analyzed for each cell sample, and the experiments were repeated twice. Colocalization analysis was performed using the Huygens Colocalization Analyzer (Scientific Volume Imaging, SVI, Hilversum, The Netherlands) to obtain the overlap coefficient value between the fluorophore pair, and to build the colocalized pixel distribution map.

### 2.6. Super-Resolution STED Microscopy

STED imaging was performed on a STED microscope platform (STEDYCON, Abberior Instruments GmbH, Göttingen, Germany) equipped with an upright Zeiss Axioimager Z.2 microscope (Zeiss Microscopy GmbH, Oberkochen, Germany) and a pulsed STED laser (Abberior Instruments GmbH) with a depletion wavelength of 775 nm working at a repetition rate of 40 MHz, a pulse duration of 1 ns, using STEDYCON SmartControl software version 9. Skin fibroblasts were immunostained using Src and lamin A/C antibodies, revealing specific goat secondary antibodies conjugated to the dyes STAR Red and STAR Orange (Abberior Instruments GmbH), respectively. STAR RED was imaged with a pulsed source at a wavelength of 640 nm (detection range between 650 and 700 nm, gated detection between 1 and 7 ns), whereas STAR Orange was excited with a pulsed source at 561 nm (detection range between 575 and 625 nm, gated detection between 1 and 7 ns). The pinhole was set to 1.1 Airy units at 650 nm. For STED imaging, an oil-immersion objective lens was used (100x Plan Apochromat oil immersion objective, 1.46 NA, Zeiss, Oberkochen, Germany). Colocalization analysis was performed using the Huygens Colocalization Analyzer (SVI) to evaluate the overlap coefficient (n = 10 of fibroblast nuclei for each sample were analyzed).

### 2.7. Fluorescence Lifetime Imaging Microscopy-FRET Analysis

Fluorescence lifetime (FLIM) imaging was carried out with a Leica TCS-SP8 FLIM (Leica Microsystems, Manheim, Germany) confocal microscope, with a time-correlated single photon counting (TCSPC) module PicoHarp 300 time-resolved unit (PicoQuant, Berlin, Germany), with a pulsed WLL tunable in the range of 470–670 nm, 2 internal SMD spectral detector channels (PMTs), through a HCX PL APO 63x/1.30 glycerol immersion CS2 objective. FLIM recordings were performed with a 40 MHz repetition rate, an image size of 256 × 256 pixels, and a zoom factor of 5x. A minimum of 1000 photons per pixel was acquired for all FLIM acquisitions. In all experiments, the laser power was adjusted to achieve average photon counting rates of ≤10^5^ photons/s and peak rates close to 10^6^ photons/s when recording FLIM images, thus preventing pile-up effects. The donor fluorescent protein, Alexa Fluor 488 (AF488), was excited with a 488 nm (laser power 3%), and fluorescence was detected by a PMT-FLIM detector in the 500–550 nm range. In the presence of the AF594 acceptor, the donor emission was detected at 550–550 nm and 600–650 nm (FRET channel). For each experiment, at least 10 individual cells were imaged, and all the experiments were repeated at least two times. Lifetime was determined in manually selected, specific regions of interest (ROIs), corresponding to two nuclear districts in which Src showed a co-distribution with lamin A/C (at the nuclear rim where lamin A/C was polymerized and in matrix regions where Src showed a dotted distribution). The best fitting results were obtained for estimating the donor fluorescence lifetime in the absence of the acceptor (τ_D_, unquenched donor lifetime), by applying a mono-exponential decay model using the n-exponential reconvolution fitting approach, and the quality of the fit was judged by the residual distribution and the goodness of the χ^2^ value (close to 1). The lifetime τ and the relative amplitude A of the individual exponential components were obtained from the SymPhoTime64 software 2.4 (PicoQuant, Berlin, Germany). By applying a bi-exponential decay, the donor fluorescence lifetime in the presence of the acceptor, using the donor lifetimes only, the values of τ_AV_ AMP (amplitude-weighted average lifetime) were obtained using SymPhoTime64 software.

The FRET efficiency (E) was calculated for each ROI using the equation:

E = 1 − (τ_AV AMP_/τ_D_), where τ_AV_ AMP is the amplitude-weighted average lifetime of the donor in the presence of the acceptor, and τ_D_ is the donor in the absence of the acceptor. The Donor-Acceptor (D-A) fluorophore distance (calculated as R-value) was obtained assuming a random orientation of both fluorophores using the following formula [27]:

$$R=R_{0}{\left(\frac{1-E}{E}\right)}^{1/6}$$

To obtain R-values, the Förster distance (R_0_) was defined as the D–A distance when E = 50% was applied. The R_0_ distance for the Alexa Fluor 488 and Alexa Fluor 594 (A-D) FRET pair was assumed to be 58.89 angstroms, as reported in the fluorescent biosensor database (FPbase FRET Calculator; https://www.fpbase.org/fret/). The tables of the images were processed using Adobe Photoshop CS4 software (Adobe Systems Inc., San Jose, CA, USA).

### 2.8. Statistical Analysis

The differences between the means of data containing two groups were analyzed using unpaired two-tailed Student’s *t*-test, and the results were considered significant when *p* < 0.05. GraphPad Prism was used for statistical analyses. Significance levels were *p* < 0.05 (*), *p* < 0.01 (**), *p* < 0.0001 (****). In the figures, error bars indicate the standard deviation of the mean (SD).

## 3. Discussion

### 3.1. Confocal and Super-Resolution Microscopy of Healthy and Laminopathic Dermal Fibroblasts

**Figure 1:**
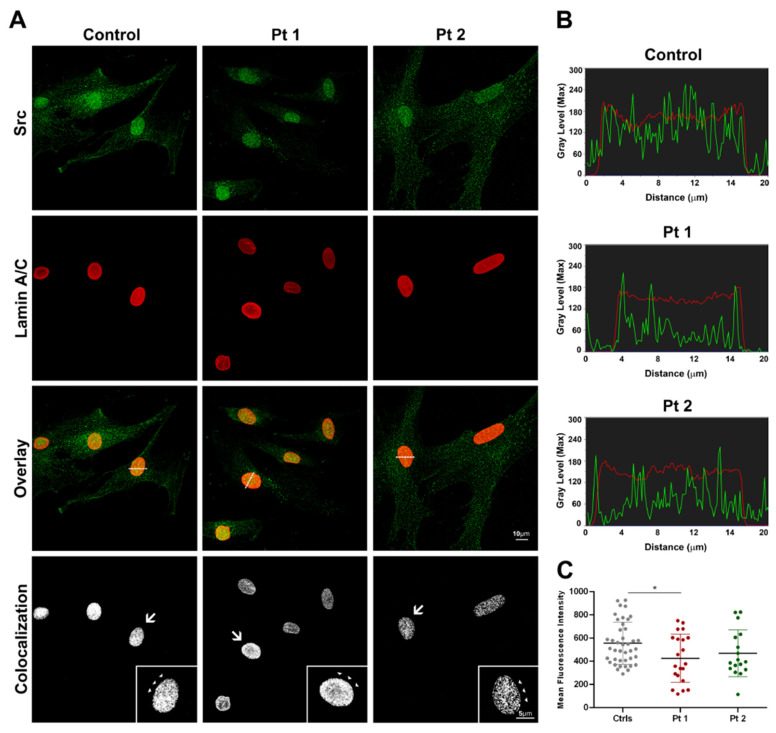
Confocal microscopy imaging of Src (in green) and lamin A/C (in red) in control and laminopathic fibroblasts. (**A**) Src immunofluorescence showed a punctate and diffuse distribution in the nuclear and cytoplasmic compartments, with a higher concentration in the nuclei, both in healthy cells and patients’ cells. Orange pixels showed the overlay of Src and Lamin A/C fluorescence in doubled-stained cells. Colocalization masks of double-stained cells (white pixels) showed the Src-lamin A/C co-distribution both at the nuclear envelope (arrows and arrowheads) and in the nucleoplasm. (**B**) Intensity line profiles of Src (green) and lamin A/C (red) across the focal central plane, as indicated by the white dotted line of representative nuclei in the overlay images. Scale bars: 10 μm for all images except insets (5 μm). (**C**) Src mean fluorescence intensity decreased in patients’ nuclei, significantly in Pt 1, compared to controls (* *p* < 0.05).

To determine the subcellular distribution of the Src protein in skin fibroblasts, we used immunofluorescence labeling with an anti-Src antibody, recognizing the total protein of cultured cells from healthy and laminopathic patients (Pt 1 and Pt 2), carrying c.1733A>T (p.Glu578Val) and c.745C>T (p.Arg249Trp) mutations in the *LMNA* gene, respectively. In all samples, a higher protein distribution in the nuclear compartment compared to the cytoplasm was detected (Figure 1A). The Src fluorescence intensity decreased in laminopathic nuclei compared to control cells, as shown by intensity line profiles across the focal central plane of reference nuclei (Figure 1B), and it was significantly reduced in patient 1 (Pt 1, *p* < 0.05; Figure 1C) cells. In double immunofluorescence, we used an antibody anti-lamin A/C that recognizes both the monomeric form, dispersed in the nucleoplasm, and the polymerized one. For each control and patient group, the mean intensity of Src fluorescence was analyzed in more than 80 nuclei, while the frequency of appearance of the dysmorphic nuclear phenotype, compared to the normal one, was observed in the lamin A/C labeling. In control cells, approximately 11% of the nuclei showed some dysmorphic signs, such as folds or blebs, while Pt 1 and Pt 2 cells exhibited a higher number of abnormal nuclei equal to 51% and 23%, respectively. In patients’ fibroblasts, Src co-distributed with lamin A/C at the nuclear rim and in the matrix district, as indicated by the corresponding colocalization masks (Figure 1A), and the differences between the overlap coefficient (OC) values were not significant (Ctrls: 0.69 ± 0.01; Pt 1: 0.69 ± 0.02; Pt 2: 0.68 ± 0.02).

In order to perform a more accurate characterization of Src and lamin A/C colocalization, we used the STED optical microscopy, a super-resolution microscopy technique capable of pushing the limit of resolution down to the nanoscale.

**Figure 2:**
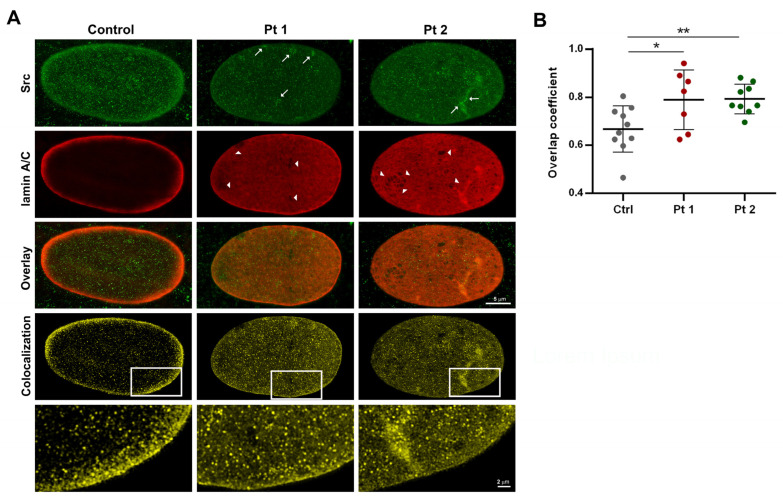
STED nanoscopy of Src and lamin A/C in nuclei from healthy and laminopathic fibroblasts. (**A**) In the healthy control nuclei, Src labeling (green) was thickened at the nuclear periphery, with a diffuse and dotted distribution in the nuclear matrix, whereas some anomalous aggregates were observed (arrows) in patients’ nuclei. Alterations in the structural organization of the lamin A/C (red) meshworks have been seen in several nuclei of the fibroblasts of patient 1 and patient 2 (arrowheads). Colocalization masks (yellow) showed the co-distribution of Src and lamin A/C at the nuclear rim in all samples and a higher concentration in the nucleoplasm of patients’ cells (high magnification of insets). Bars: 5 µm and 2 µm. (**B**) Mean values of the overlap coefficient quantified in STED images of double-stained fibroblast nuclei. (** *p* < 0.01; * *p* < 0.05).

By STED imaging, we observed a fine punctate and diffuse distribution of Src protein inside healthy nuclei, with a higher signal intensity at the periphery, where the lamin A/C signal was concentrated (Figure 2A). In laminopathic nuclei, Src localization was comparable to that of controls, although with a more reduced intensity, especially at the nuclear rim; furthermore, some anomalous Src aggregates or clusters were noticed (Figure 2A). Lamin A/C labeling was particularly concentrated at the periphery of healthy nuclei, as expected; in laminopathic cells, a wide lamin A/C distribution in the nuclear matrix and abnormalities in the microfilamentous meshwork texture were noticed in several nuclei (Figure 2A). Super-resolved Src-lamin A/C colocalization masks highlighted a higher degree of overlap in Pt 1 (OC: 0.789 ± 0.04; *: *p* < 0.05) and Pt 2 (0.793 ± 0.02; **: *p* < 0.01) nuclei compared to the controls (0.668 ± 0.03; Figure 2A,B).

### 3.2. FLIM and FRET-FLIM Microscopy in Healthy and Laminopathic Fibroblasts

**Figure 3:**
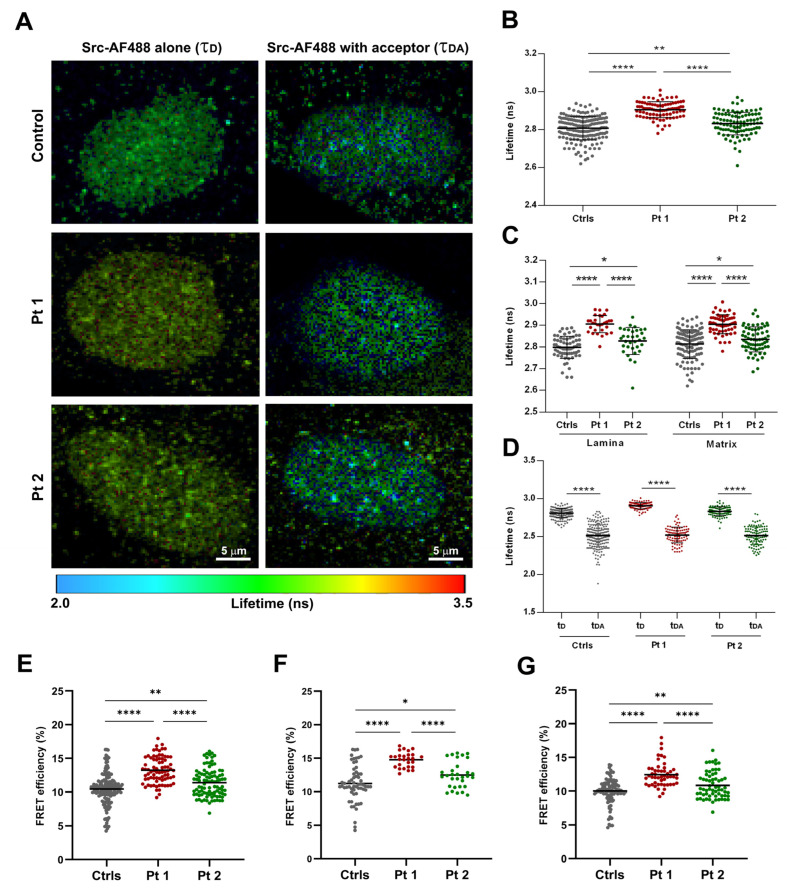
FLIM and FLIM-FRET microscopy of Src and lamin A/C in healthy and laminopathic fibroblast nuclei. (**A**) Fluorescence lifetime imaging of Src-AF488 donor in the absence (τ_D_, left panel) or in the presence (τ_DA_, right panel) of a lamin A/C-AF594 acceptor. Different τ values were visualized via color code lifetime scale bar (from 2.0 to 3.5 ns of range). (**B**) Src-AF488 mean lifetime values in the absence of the acceptor (τ_D_) showed a significant increase in laminopathic nuclei compared to controls (**** *p* < 0.0001 in Pt 1; ** *p* < 0.01 in Pt 2). (**C**) The statistical analysis of the Src-AF488 mean τ_D_ values in the two specific ROIs revealed significant lifetime changes between controls and patients’ nuclei, both in the lamina and in the nuclear matrix regions (**** *p* < 0.0001; * *p* < 0.05). (**D**) Src-AF488 donor lifetime in the presence of the acceptor molecule (τ_DA_, amplitude weighted lifetime) was significantly decreased in all samples, with a greater extent in patients’ fibroblasts (**** *p* < 0.0001). (**E**–**G**) Quantified FRET efficiency values (mean ± sem) of the Src-AF488 and lamin A/C-AF 594 pair obtained in all selected ROIs (**E**), at the nuclear rim (**F**) and in the nucleoplasm (**G**) in controls (gray dots), in Pt 1 (red dots) and Pt 2 (green dots) nuclei (**** *p* < 0.0001; ** *p* < 0.01; * *p* < 0.05).

To study Src–lamin A/C proximity and interaction, we used a fluorescence-decay-kinetics method based on the detection of the donor fluorescence lifetime variations, in the presence and absence of the acceptor fluorophore (AF594). In single-labeled samples, the mean fluorescence lifetime (τ) of the fluorophore conjugated to the Src antibody (AF488) was detected by time-domain FLIM on a pixel-by-pixel basis. FLIM measurements were calculated in the two specific selected ROIs: at the nuclear rim and in matrix regions where Src showed a dotted distribution (Figure 3A). The FLIM data of the Src-AF488 alone (τ_D_) were visualized in the lifetime image (Figure 3A), wherein each pixel was represented with a color code corresponding to a specific τ value (in nanoseconds, as indicated in the scale bar).

Donor lifetime values were increased in patients’ nuclei compared to healthy samples in the two regions of interest (Figure 3B,C), suggesting a different balance between bound and unbound forms of Src. Furthermore, inside each group, τ_D_ values in the nuclear periphery and nucleoplasm (Table S1) were not different.

In double stained samples, in the presence of lamin A/C-AF594 acceptor, the lifetime of the Src-AF488 donor (amplitude weighted lifetime, τ_DA_) reduced significantly in controls and patients’ nuclei (*p* < 0.0001; Figure 3D), with a greater decrease in Pt 1 (*p* < 0.0001) and to a lesser extent, in Pt 2 (*p* < 0.0001), as confirmed by the calculated FRET efficiencies (Figure 3E–G). The rate of energy transfer (E) in total ROIs was greater in Pt 1 (13.25 ± 0.22) compared to Pt 2 (11.39 ± 0.22), and both of them were significantly different from the control mean value (10.45 ± 0.18, *p* < 0.0001 vs. Pt 1; *p* < 0.01 vs. Pt 2; Figure 3E; Table S2). In all samples, a higher value of interaction between Src and lamin A/C was observed at the level of the nuclear rim (Figure 3F) compared to the nucleoplasm (Figure 3G). By measuring the donor lifetime in the presence and the absence of the acceptor, we calculated the distance (RDA) between the donor-AF488 and acceptor-AF594 labeled proteins. The higher transfer efficiencies detected in patients’ nuclei, particularly in Pt 1 (Figure S1) were indicative of a shorter RDA between the chromophores engaged in FRET (Table S3). Furthermore, a reduced RDA between Src and lamin A/C was observed at the nuclear periphery compared to the nucleoplasm in Pt 1 (*p* < 0.0001) and Pt 2 (*p* < 0.001), whereas in healthy cells, no differences were observed (Table S3).

In this study, we demonstrated the relationship between lamin A/C and Src proteins in healthy and laminopathic fibroblasts at a nanoscale level, by advanced imaging-based microscopy techniques. The two patients assessed showed a different clinical phenotype, an atypical progeroid Werner’s syndrome (Pt 1), and a congenital muscular dystrophy (Pt 2), respectively, as a consequence of missense mutations in different domains of the *LMNA* gene.

Using confocal and super-resolution STED microscopy and molecular imaging tools, such as the FLIM-FRET analysis, we provided evidence of the differential Src distribution and activity/activation represented by an altered lifetime for Src-tagged Alexa Fluor 488 fluorophore in the nuclear compartment of skin fibroblasts in comparison to normal cells. This finding suggests a diverse conformational state of Src tyrosine kinase [28], likely influencing its activation in laminopathic nuclei. Moreover, the altered pattern of subcellular localization and protein conformation of Src in laminopathic cells could represent a new diagnostic marker for these pathologies.

In our study, we provide, for the first time, evidence of a close relationship between Src and lamin A/C at the level of the nuclear lamina and in the nucleoplasm of both healthy and laminopathic fibroblast nuclei. Indeed, we obtained more solid results of energy transfer efficiency thanks to the use of the FRET-FLIM technique that takes advantage of the lifetime’s independence from variations in donor fluorophore concentration. The higher values of transfer efficiency detected in laminopathic nuclei compared to healthy cells may be suggestive of an aberrant interaction between Src and lamin A/C, and further studies will be needed to uncover whether this depends mainly on the phosphorylation activity of Src, not only at the level of the nuclear lamina but also in the nucleoplasm districts, where the mechanical role of lamin A is well known [29]. In addition, the evaluation of the FRET efficiencies in FLIM-FRET experiments demonstrated a statistically significant difference between healthy and laminopathic cells, but also between the two patients, with different forms of laminopathies, thereby suggesting this interaction as a suitable diagnostic and prognostic marker.

## 4. Conclusions

In conclusion, this work demonstrates, for the first time, a molecular interaction between Src and lamin A/C in the nuclear compartment of laminopathic fibroblasts. Despite the very small number of patients assessed, due to the rarity of these pathologies and the limited biological material available for molecular and cellular studies, the assessment of these two patients was sufficient to provide clear evidence of an aberrant and, potentially, pathogenetic interaction between Src and lamin A/C. It is evident that future work is needed to further characterize the described molecular interaction between Src and lamin A/C in laminopathic patients in order to shed light on the effects mediated by the *LMNA* gene-specific mutations on the enzymatic activity of Src tyrosine kinase. Therefore, the extension of this study to a larger number of *LMNA* mutated patients with different clinical phenotypes will further provide pivotal elements on the regulatory role played by Src in laminopathies, thereby definitively opening new diagnostic and therapeutic approaches for patients.

## Data Availability

The original contributions presented in this study are included in the article/Supplementary Materials. Further inquiries can be directed to the corresponding author(s).

## Supplementary Materials

The supporting information can be downloaded at: https://www.mdpi.com/article/10.3390/ijms252413365/s1.
