# Supplementary material for: Imaging-Based Molecular Interaction Between Src and Lamin A/C Mechanosensitive Proteins in the Nucleus of Laminopathic Cells
Source: Int J Mol Sci. 2024 Dec 13;25(24):13365. doi: 10.3390/ijms252413365 (PMC11678420; doi:10.3390/ijms252413365)
Supplement: Supplementary file 1 [file ijms-25-13365-s001.zip › ijms-3336952-supplementary.pdf]

Supplementary Figures and Tables

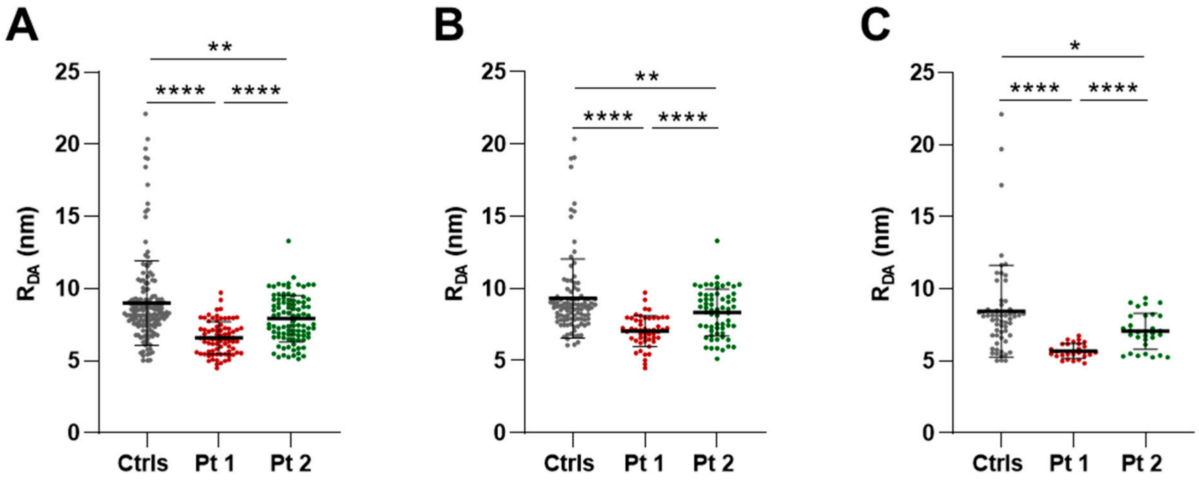

Supplementary Figure S1 – Quantification of the R_{DA} intermolecular distance obtained by FRET-FLIM measurements in all ROIs (**A**), at the nuclear periphery (**B**) and in the nucleoplasm (**C**) in healthy (Ctrls), Pt 1 and Pt 2 cells. ****$p < 0.0001$; **$p < 0.01$; *$p < 0.05$.

Supplementary Table S1 - Fluorescence lifetime data in stained cells with Src-AF488 alone (τ_D) and in presence of lamin A/C-AF594 acceptor (τ_{DA}), are expressed in nanoseconds (ns) as mean ± sem.

Sample	τ	Total ROIs	Lamina	Matrix
Ctrls	τ_D	2.807 ± 0.01	2.80 ± 0.01	2.81 ± 0.01
	τ_{DA}	2.510 ± 0.01	2.50 ± 0.01	2.53 ± 0.01
Pt 1	τ_D	2.905 ± 0.004	2.91 ± 0.01	2.90 ± 0.01
	τ_{DA}	2.519 ± 0.01	2.47 ± 0.01	2.55 ± 0.01
Pt 2	τ_D	2.832 ± 0.006	2.827 ± 0.011	2.834 ± 0.007
	τ_{DA}	2.511 ± 0.01	2.48 ± 0.02	2.53 ± 0.01

Supplementary Table S2 - FRET efficiencies (E) measurements obtained from FRET-FLIM experiments are expressed in % values as mean ± sem. The FRET Efficiency data were significantly higher at the level of the nuclear periphery compared to the nucleoplasm in control samples ($p < 0.01$)(1), as well as in Pt 1 ($p < 0.0001$)(2) and in Pt 2 ($p < 0.002$)(3) nuclei.

	**FRET Efficiency (%)**		
**Sample**	**Total ROIs**	**Nuclear rim**	**Matrix**
**Ctrls**	10.45 ± 0.18	11.22 ± 0.36(1)	10.00 ± 0.19
**Pt 1**	13.25 ± 0.22	14.76 ± 0.22(2)	12.45 ± 0.25
**Pt 2**	11.39 ± 0.22	12.49 ± 0.35(3)	10.85 ± 0.25

Supplementary Table S3 – Mean R_{DA} values obtained from FRET-FLIM experiments were expressed in mean ± sem. The R_{DA} intermolecular distance was significantly shorter at the level of the nuclear periphery compared to nuclear matrix in Pt 1 ($p < 0.0001$)(1) and in Pt 2 ($p < 0.001$)(2) nuclei.

	**R_{DA} (nm)**		
**Sample**	**Total ROIs**	**Nuclear rim**	**Matrix**
**Ctrls**	9.00 ± 0.23	8.45 ± 0.42	9.32 ± 0.27
**Pt 1**	6.59 ± 0.13	5.71 ± 0.10 (1)	7.05 ± 0.15
**Pt 2**	7.92 ± 0.17	7.06 ± 0.22 (2)	8.34 ± 0.20
